# Inter-hospital communication and transfer practices during COVID-19 Pandemic in Karachi, Pakistan. A brief overview

**DOI:** 10.12669/pjms.36.COVID19-S4.2803

**Published:** 2020-05

**Authors:** Saima Salman, Syed Ghazanfar Saleem, Adeel Khatri, Imran Jamal, Quratulain Maroof, Ashar Alam

**Affiliations:** 1 Saima Salman, Emergency Department, The Indus Hospital, Karachi, Pakistan; 2 Syed Ghazanfar Saleem, Emergency Department, The Indus Hospital, Karachi, Pakistan; 3 Adeel Khatri, Emergency Department, The Indus Hospital, Karachi, Pakistan; 4 Imran Jamal, Emergency Department, The Indus Hospital, Karachi, Pakistan; 5 Quratulain Maroof, Emergency Department, The Indus Hospital, Karachi, Pakistan; 6 Ashar Alam, Medical Directorate, The Indus Hospital, Karachi, Pakistan

**Keywords:** COVID 19, Pandemic, Referral systems, Emergency care

## Abstract

**Objective:**

To discuss the referral mechanisms established for safe and expeditious inter-facility transfer of COVID 19 positive patients to ensure their referrals through establishing proper communication channels.

**Methods:**

Mobile phone and WhatsApp based groups, administrated by The Indus Hospital were established in April 2020. Through detailed reports and frequent communication, factors like bed and ventilator availability across these facilities are shared. Weekly reports through zoom meetings updating the key stake holders, discussion of problems faced and planning for the week ahead are also done.

**Result:**

The establishment of these groups has been successful in ensuring referral to and from The Indus Hospital to various healthcare facilities across Karachi using appropriate ambulance services.

**Conclusion:**

The development of referral mechanisms is the need of the day that has been highlighted through the COVID 19 pandemic. It is our hope that these mechanisms are sustained after the pandemic and result in improvement in patient outcome through proper referrals.

## INTRODUCTION

COVID-19 (Novel Coronavirus-19) is a new strain of coronavirus that had previously not been identified in humans. It first emerged in Wuhan, China, in December 2019, and has since been declared as an outbreak by World Health Organization (WHO). The global burden of disease is 3,349,786 with a mortality of 238,628 till date.^1^

In addition to the extensive planning required to cater to patients with COVID 19 in hospitals as well as instructions for home quarantine in stable patients, the undertaking also involves establishment of state run quarantine facilities, establishment of protocols for surge capacity and liaison between healthcare facilities and government for proper patient management.^2^,^3^ With Pakistan facing double burden of disease under normal environmental conditions and total expenditure on health just 2.6% of the Gross domestic product (GDP), the situation calls for a wise utilization of existing resources and prompt decision making on part of healthcare officials in Emergency departments (ED) as well as inpatient admissions.^4^-^6^ Informal and haphazard communication and transfer processes may result in delays in care, inappropriate triage, overcrowding at tertiary hospitals, and compromised patient outcomes.^7^ The knowledge gaps in COVID 19 and ever evolving disease pattern with its varied presentation has made the patients’ ED disposition very challenging. Adding on to this problem is the lack of centralized Emergency Medical Services (EMS), absence of systematic referral systems that result in delays, improper referrals to hospitals lacking the desired facilities and unnecessary exposure of healthcare workers in this delayed undertaking.

## METHODS

Keeping in mind the problems related to referrals of COVID 19 patients with our limited resources and challenges in Karachi, mobile phone and WhatsApp based groups called, “COVID unit Coordination” and a subgroup “COVID Karachi Daily updates”, were established that became operational on 20 April, 2020, with six hospitals on board. These groups are administrated by The Indus Hospital (TIH) with an Excel based dashboard and collaborative institutes include The Aga Khan Hospital, Sindh Institute of Urology and Transplant (SIUT), Civil Hospital Karachi, Ojha campus of Dow University Hospital and Jinnah Post graduate Medical Centre (JPMC). The line is manned 24/7 and updates by all stake holders are frequently posted. Focal persons have been nominated form all hospitals, ranging from Medical directors, superintendents and Administrative leads in Critical care units and EDs. A detailed report is shared every morning comprising of variables like available hospital beds and number of ventilated and non-ventilated patients. Since all hospitals are functioning on 100% occupancy during the pandemic, frequent referrals need to be made as soon as beds become vacant. This is facilitated expeditiously through text messages and direct phone calls, all the while ensuring the availability of desired patient needs. A detailed report of this activity is shared on weekly basis with all the stake holders with weekly zoom meetings, updating the key stake holders, discussing the problems faced and planning for the week ahead.

Another step taken is the establishment of liaison with EMS, Aman and Edhi Ambulance services. Although no written Standard Operative Procedures (SOPs) exist, Aman ambulances have been designated by the government for the inter-hospital transport of all COVID patients, whether stable or unstable, after arrangements have been made through the COVID groups. The dead bodies of the suspected COVID deceased patients are transported by Edhi ambulance with clear burial instructions.

## RESULTS

Since the inception of the groups, nineteen patients have been referred from The Indus Hospital to various other facilities. All the referred patients were COVID 19 positive, tested through nasophyrangeal swabs and PCR. Three patients were intubated and sixteen patients required just high flow oxygen. One patient had renal compromise and had to be dialysed. All nineteen patients underwent investigations including CBC, Chest X ray, renal profile and blood cultures and received broad spectrum antibiotics with methyl prednisolone. All the cases were discussed with Infectious Diseases department at TIH prior to their referrals. These patients were referred out after clear communication regarding their ventilator needs and COVID related complications by TIH team. Three patients were referred to SIUT, seven to Civil hospital, three to Ojha campus of Dow University of Health Sciences, five to JPMC and one to The Aga Khan hospital.

Similarly, five patients were received from other facilities and were admitted at TIH in established COVID inpatient according to their requirements in Intensive care units, high dependency units and ward setup.

Verbal feedback was taken from the receiving facilities post referral and safe referral was ensured. Verbal feedback was also taken from the patients and attendants prior to their transfer and they also expressed their satisfaction, knowing that they were being shifted to a facility, catering to their needs.

## DISCUSSION

There is ample opportunity to strengthen referral systems and practices in Pakistan. The Pakistani health system consists of public, private for-profit, and private non-profit institutions that vary greatly in their capabilities in managing the burden of COVID 19 pandemic. Many institutes have stepped up during this time with the establishment of designated COVID emergencies consisting of respiratory and non respiratory emergencies along with inpatient and Intensive care units (ICUs) across the country. Government led initiatives have included establishment of web pages with regular updates,^8^ social, electronic and print media based campaigns to create awareness regarding social distancing and regular hand washing with enforcing lockdown.

According to National Disaster Management Authority (NDMA)of Pakistan till May 6^th^, the total number of confirmed cases of COVID19 in Pakistan were 22,550, total deaths were 526 and the number of cases who have recovered were 6,217. The number of confirmed cases in Sindh province till May 6^th^ were 8189 9 Pakistan has also become the 25^th^ country in the world which has recorded over five hundred deaths from COVID19. The capital of Sindh, Karachi has an estimated population of 14.91 million.^10^ many slum areas with people living in close quarters and variation in scope of healthcare facilities. The need for expeditious transfer of patients among various hospitals has always been voiced at various forums but no measures have been taken so far in the form of well-defined pathways and SOPs, neither between hospitals nor at the level of policy makers. In situations of mass disasters, natural and manmade, this need becomes all the more visible. The current pandemic of COVID 19 has established this fact, yet once again.

**Fig.1:**
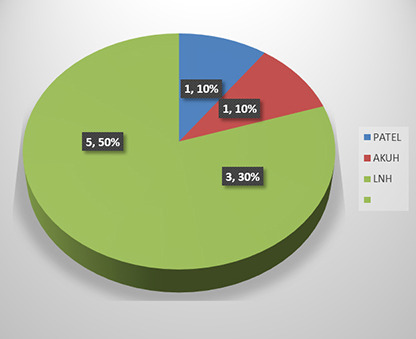
Received from other hospital.

Through establishment of mobile phone and WhatsApp based informal referral system, the transfer of very sick, infectious patients has been found to be expeditious and safe. The liaison between transferring facilities ensures that preparations are made before the arrival of the patient by the receiving facility and delays are minimized.

Another facet for improvement that has been brought into play through this exercise is the judicious use of ambulance services in transferring patients to the appropriate facilities, based on the expertise and capacity of the ambulance.

All these endeavors have the potential to be scaled up to be continued once the pandemic is contained as proper referral systems and EMS are the need of the day in a busy city like Karachi.

**Fig.2:**
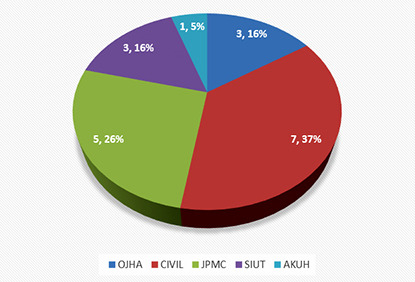
Referred to other hospitals.

### Authors’ Contribution

**SS, SGS, IJ, QM:** Conceived, wrote and did literature search.

**AK:** Data collection and feedback.

**AA:** Editing and proof reading of manuscript.
